# Assessment of micromotion at the bone-bone interface after coracoid and scapular-spine bone-block augmentation for the reconstruction of critical anterior glenoid bone loss—a biomechanical cadaver study

**DOI:** 10.1186/s12891-023-06914-9

**Published:** 2023-10-05

**Authors:** Yasmin Youssef, Martin Heilemann, Peter Melcher, Jean-Pierre Fischer, Stefan Schleifenbaum, Pierre Hepp, Jan Theopold

**Affiliations:** 1 Department of Orthopaedic Surgery, Traumatology and Plastic Surgery, University of Leipzig Medical Center, Leipzig, Germany; 2 ZESBO—Center for Research on Musculoskeletal Systems, Leipzig University, Leipzig, Germany

**Keywords:** Micromotion, Primary stability, Bone-block augmentation, Glenoid, Spina-scapula bone block

## Abstract

**Background:**

Glenoid bone loss is among the most important risk factors for recurrent anterior shoulder instability, and a bony reconstruction is recommended in cases of critical bone loss (> 15%). The commonly used surgical techniques, including coracoid transfer, are associated with considerable complications. The aim of this study was to assess the motion at the glenoid-bone-block interface after coracoid and spina-scapula bone-block reconstruction of the anterior glenoid.

**Methods:**

Twelve cadaveric shoulders were tested. A 20% bone defect of the anterior glenoid was created, and the specimens were randomly assigned for glenoid augmentation using a coracoid bone block (n = 6) or a scapular spine bone block (n = 6). The glenoid-bone interface was cyclically loaded for 5000 cycles with a force of 170 N. The micromotion was tracked using an optical measurement system (GOM ARMIS) and was evaluated with the GOM Correlate Pro software.

**Results:**

The most dominant motion component was medial irreversible displacement for the spina-scapula (1.87 mm; SD: 1.11 mm) and coracoid bone blocks (0.91 mm; SD: 0.29 mm) (n.s.). The most medial irreversible displacement took place during the first nine cycles. The inferior reversible displacement was significantly greater for spina-scapula bone blocks (0.28 mm, SD: 0.16 mm) compared to coracoid bone blocks (0.06 mm, SD: 0.10 mm) (p = 0.02).

**Conclusions:**

The medial irreversible displacement is the dominant motion component in a bone-block reconstruction after a critical bone loss of the anterior glenoid. The spina-scapula and coracoid bone blocks are comparable in terms of primary stability and extent of motion. Thus, spina-scapula bone blocks may serve as alternatives in bony glenoid reconstruction from a biomechanical point of view.

## Background

Glenoid bone loss is one of the most important risk factors for recurrent anterior shoulder instability [13, 29]. The critical bone loss of the anterior glenoid was described as a bone loss of over 15% of the glenoid’s total surface area [21–23]. In cases of extensive bone defects of the anterior glenoid, reconstruction techniques using bone blocks are recommended [5, 16]. The coracoid autografts (Latarjet procedure), iliac crest autografts, and allografts are common bone blocks [5, 16]. However, all bone blocks have limitations and complication rates as high as 30% reported, particularly after a failed soft tissue repair [7]. The Latarjet procedure, especially, has been associated with considerable surgical complications due to its proximity to neurovascular structures [8, 14]. Similarly, the iliac-crest bone block has been associated with significant complications like infection, pain, immobility, and nerve injury at the iliac site [10, 15, 19].

Recently, the scapular spine has been described as a potential alternative source for bone-block autografts in anterior glenoid reconstruction [17, 26]. The advantages include proximity to the surgical site, good anatomical accessibility, minimal muscular attachment, and the absence of neurovascular structures [4, 24, 25]. Furthermore, Rohman et al. have shown that the dimensions of scapular-spine bone blocks are comparable to those of coracoid and iliac-crest bone blocks [18]. An arthroscopic glenoid reconstruction using an autologous scapular-spine bone block has been described [17, 26]. In a case series of 27 patients, Xiang et al. demonstrated that autologous scapular spine blocks could achieve satisfactory results in a short-term follow-up in subcritical glenoid bone loss [26]. In those patients, neither age, sex, body mass index, nor smoking were correlated with graft resorption after arthroscopic reconstruction with a scapular-spine bone block [28].

The primary stability and micromotion at the bone-block glenoid interface could influence the reconstruction outcome in terms of biomechanical stability and clinical functionality. Therefore, the aim of this study was to assess the motion at the glenoid-bone-block interface after coracoid and spina-scapula bone block reconstruction of the anterior glenoid with critical bone loss.

## Methods

### Specimen preparation

Twelve ethanol-glycerin fixed cadaveric shoulder specimens, 6 right and 6 left shoulders (unmatched), were obtained from the Institute of Anatomy. The specimens were stored at 4 °C and examined before preparation to ensure that there were no severe degenerative changes of the glenoid. The soft tissue was removed from the specimens, leaving only the bony components of the shoulder (scapula, acromion, glenoid, and coracoid).

### Ethical considerations

All donors originated from the Institute of Anatomy of the University of Leipzig and had given written informed consent to dedicate their bodies to medical education and research purposes. Being part of the body donor program regulated by the Saxonian Death and Funeral Act of 1994 (3rd section, paragraph 18, item 8), institutional approval for the use of the post-mortem tissues of human body donors was obtained. The authors declare that all experiments were performed according to the ethical principles of the Declaration of Helsinki. The institutional approval for the use of the post-mortem specimens was obtained; hence, approval by the ethics committee was not required.

### Graft preparation

The graft preparation and surgical reconstruction were performed by an experienced shoulder surgeon to ensure consistency. A 20% bone defect of the anterior glenoid was created to simulate critical bone loss. The specimens were randomly assigned into two groups:

- Group 1: anterior glenoid augmentation using a coracoid bone-block (n = 6).
- Group 2: anterior glenoid augmentation using a scapular-spine bone-block (n = 6).

All bone blocks were fixed using two 4 mm long cannulated screws (DePuy Synthes, Zuchwill, Switzerland) after placing 2.7 mm quatricortical boreholes. The specimens were embedded in a mounting tray using epoxy resin, with the glenoid surface aligned parallel to the base plate.

### Biomechanical testing

**Fig. 1:**
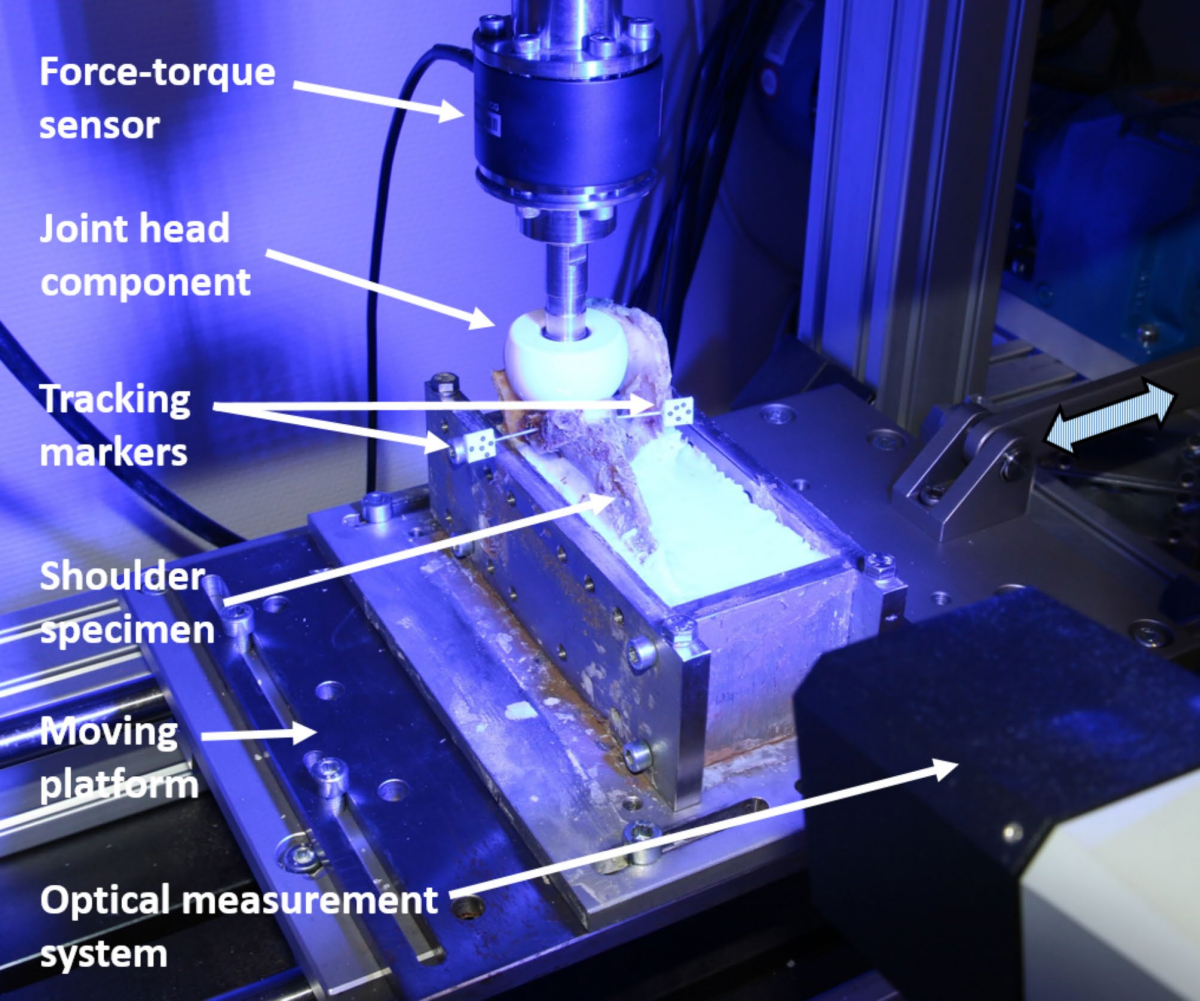
Test setup: Specimen with attached markers for optical micromotion measurement in the mounting tray and aligned head component

All specimens underwent cyclic testing using a custom-built test bench (Fig. 1). This setup was based on the ‘rocking horse’ setup previously described by the ASTM (American Society for Testing and Materials) F2028 for the biomechanical assessment of glenoid loosening. The setup allows constant force transmission between an oscillating ceramic head component, simulating the humerus head, onto the reconstructed glenoid. A vertical force of 170 N was applied onto the glenoid surface, which was adjusted using a 6-axis force-torque sensor (ME-Messsysteme GmbH, Hennigsdorf, Germany; uncertainty: ±1 N). The horizontal movement of the ceramic head component across the glenoid surface was executed by a three-phase motor. Each specimen was aligned in the test bench considering the two oscillation end-positions of the ceramic head component on the reconstructed glenoid surface. In the first position, the centre of the head component was aligned so that it only loaded the original glenoid surface; in the second end position, it only loaded the bone block. Thus, the augmented bone block was loaded and unloaded in each cycle. A complete test comprised 5000 cycles at a frequency of 1 Hz.

The movements at the glenoid-bone-block interface were recorded using two markers attached to the glenoid and bone block. The marker positions were tracked using an optical three-dimensional measurement system (ARMIS 3D CAMERA, Carl Zeiss GOM Metrology GmbH, Braunschweig, Germany). The marker motion was registered with a frame rate of 25 Hz at five defined cycle complexes (cycles 5, 500, 1000, 2500, and 4995). Each cycle complex, therefore, comprised nine consecutive cycles, four before and after the eponymous cycle, respectively.

The recorded data were evaluated with the digital image correlation software *GOM Correlate Pro* (Carl Zeiss GOM Metrology GmbH, Braunschweig, Germany). The interface between the bone block and glenoid was set as the origin of the coordinate system. Six degrees of freedom analysis were performed, and translational components of motion (uncertainty: 0.01 mm) were exported for further data analysis.

### Statistical analysis

Data processing and statistical analysis were performed using Matlab R2019a (MathWorks, Natick, Massachusetts, USA). Micromotion data was categorised into irreversible (irrevocable translation of the block) and reversible displacement (spatial oscillation of the block due to cyclic loading) and compared between the two surgical procedures using an independent two-tailed t-test. The statistical significance was set at p < 0.05.

## Results

None of the augmented bone blocks failed (complete loosening) during testing. In both groups, the medial irreversible displacement was the most dominant micromotion component at the glenoid-bone-block interface. The mean medial irreversible displacement was 1.87 mm (standard deviation (SD): 1.11 mm) for the spina-scapula block and 0.91 mm (SD: 0.29 mm) for the coracoid block. There was no significant difference between the two groups (p = 0.07). The greatest proportion of the medial irreversible displacement occurred during the first nine loading cycles, with a mean of 1.05 mm (SD: 0.50 mm) for the spina-scapula blocks and 0.58 mm (SD: 0.31 mm) for the coracoid bone blocks (p = 0.08).

The inferior reversible displacement was significantly greater for the spina-scapula bone blocks (0.28 mm, SD: 0.16 mm) compared to the coracoid bone blocks (0.06 mm, SD: 0.10 mm) in the final cycle complex (p = 0.02); however, it was the least dominant motion component, when considering the absolute values. No significant differences were identified for any other micromotion directions and components (irreversible/reversible).

**Fig. 2:**
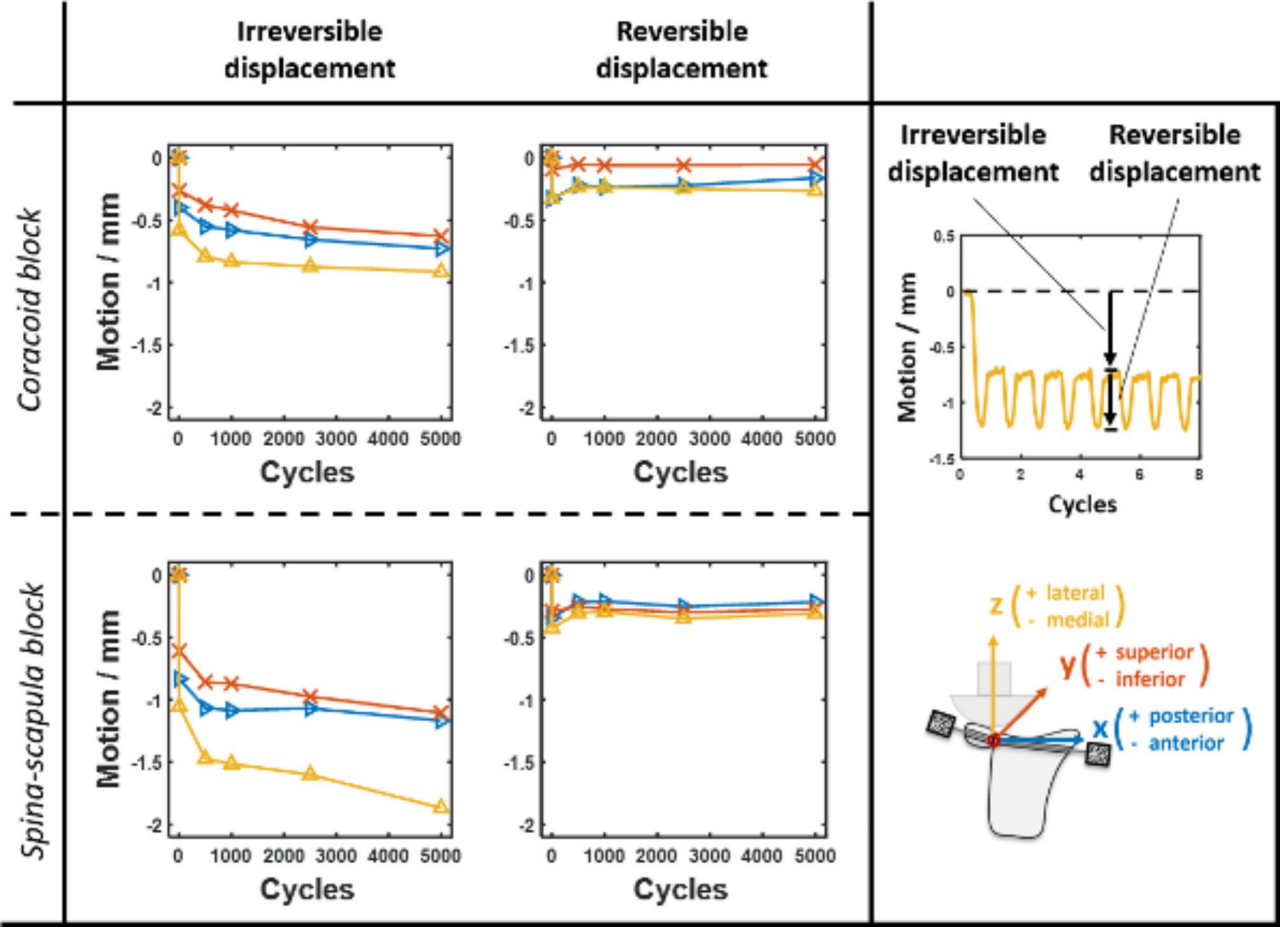
Irreversible and reversible displacements between coracoid and spina-scapula bone blocks and glenoid during testing

Figure 2 shows the mean irreversible and reversible displacements for both study groups. The mean final displacement and corresponding standard deviation after the completed 5000 cycles are presented in Table 1.

**Table 1:** Mean irreversible and reversible displacement between coracoid and spina-scapula bone block and glenoid after complete testing (* indicates a significant difference between both bone block types)

		*Spina-scapula block*			*Coracoid block*	
mean ± standard deviation		**irreversible displacement**	**reversible displacement**		**irreversible displacement**	**reversible displacement**
	**x**	-1.17 ± 0.65	-0.22 ± 0.19	**x**	-0.73 ± 0.57	-0.16 ± 0.30
	**y**	-1.10 ± 0.59	-0.28 ± 0.16 *	**y**	-0.63 ± 0.25	-0.06 ± 0.10 *
	**z**	-1.87 ± 1.11	-0.31 ± 0.20	**z**	-0.91 ± 0.29	-0.27 ± 0.20

## Discussion

The aim of this study was to determine the primary stability and motion at the glenoid-bone-block interface after coracoid and spina-scapula bone-block reconstruction of the anterior glenoid with critical bone loss. It could be shown that medial irreversible displacement is the dominant motion component in bone-block reconstruction after critical bone loss of the anterior glenoid and that spina-scapula and coracoid bone blocks are comparable in terms of primary stability and extent of micromotion.

Choosing an optimal bone graft is essential in the reconstruction of the anterior glenoid with critical bone loss to ensure a good functional and clinical outcome and to avoid donor-site morbidity. Coracoid bone blocks and iliac crest autografts are commonly used [5, 17]; however, they are associated with considerable complication rates, which can be as high as 30% [7, 14, 20], especially the Latarjet procedure with complications such as neurovascular injuries [8, 14]. This can be explained by the proximity of the brachial plexus nerves and vessels to the surgical site [12]. Scapular spine blocks could be used as alternative bone blocks in anterior glenoid augmentation. Previous studies have shown that the anatomic dimensions of scapular spine blocks are comparable to those of coracoid and iliac crest bone blocks [18]. The advantages of the scapular spine are its proximity to the surgical site, good anatomical accessibility, minimal muscular attachment, and the absence of neurovascular structures, including injuries of the axillary, suprascapular, and musculocutaneous nerves [4, 24, 25]. This could facilitate preparation and surgery and could minimise surgical site morbidity. The scapular spine has already been used in facial reconstruction with minimal harvest site complications [2, 24, 25]. The techniques for arthroscopic glenoid reconstruction using scapular bone blocks have been described [9, 17, 26]. Further, in a short-term follow-up, Xiang et al. demonstrated that satisfactory results could be achieved [26].

The scapular spine block offers potential advantages during surgery, as described above. However, the biomechanical suitability of the scapular spine block must be ensured. The quantification of reversible and irreversible micromotion in three dimensions at the glenoid-bone-block interface provided insight into the biomechanical behavior at the interface. A modified version of the ‘rocking horse’ setup, as defined by the ASTM F2028 for the testing of glenoid loosening, was used in this study to evaluate the motion at the glenoid-bone-block interface [6]. The cyclic loading of the bone block using a ceramic head component was used to simulate the movement and force of the humerus on the glenoid surface. A force of 170 N was chosen, as Bergman et al. have demonstrated that the glenohumeral contact force during glenohumeral flexion and abduction can be up to 170 N in the medial direction, even before any perceptible motion of the joint [1]. Therefore, despite temporary post-operative immobilisation after bone-block augmentation of the glenoid, forces act on the glenoid-bone-block interface.

At the bone block two different types of motion can be observed—reversible and irreversible displacement. While irreversible motion refers to any translation of the bone block, which is irrevocable, reversible displacement refers to the spatial oscillation of the block due to cyclic loading. Both motion components are schematically depicted in the upper right corner of Fig. 2. This study revealed that medial irreversible displacement is the dominant motion at the glenoid-bone-block interface after bony glenoid reconstruction using coracoid and spina-scapula bone blocks. More than 50% of the final medial irreversible displacement occurred in the first nine cycles and plateaued within the first 500 cycles. This suggests that there is an initial setting behavior of the bone block in the first loading cycles, after which the irreversible replacement becomes less prominent. Comparing both bone blocks, no significant difference in irreversible displacement was observed. However, the inferior reversible displacement was significantly greater for spina-scapula bone blocks compared to coracoid bone blocks in the final cycle complex. The inferior displacement was the least dominant micromotion component in both groups (0.28 mm, SD: 0.16 mm in the spina-scapula bone blocks compared to 0.06 mm, SD 0.10 mm in the coracoid bone blocks) in the final cycle complex. These motion dimensions could be negligible in clinical routine for the primary stability of the bone block.

In vivo bone healing is a complex process influenced by many patient-related (e.g., bone density) and external factors, including the mechanical environment [3]. Even if there are still no established cut-offs for the extent of accepted micromotion at fracture sites, dynamic fixation of bone fragments that allow some movement at the bone-bone interface seems to promote callus formation [3, 27]. Therefore, our study results suggest that coracoid and spina-scapula bone blocks are biomechanically comparable when considering the micromotion at the glenoid-bone-block interface.

A previous study by Kuan et al. also revealed that coracoid and spina-scapula bone blocks have comparable biomechanical behavior in terms of construct stiffness, average failure load, and cyclic displacement [11]. Kuan et al. described a loading scenario; however, in this study the focus was specifically the extent of micromotion at the glenoid-bone-block interface and cyclic anteroposterior motion between the glenoid and head component.

This study has some limitations. First, only a small sample size was considered for each surgical group due to the limited availability of donor shoulders. As only 12 shoulder specimens were available, no sample size calculation was performed. Second, the experimental setup only represents a simulated setting involving only the bony components of the shoulder. Therefore, the results can only act as a reference as they may not fully represent the biomechanical in vivo circumstances where the soft-tissue components, including muscles, tendons, and fascia, affect the biomechanical stability of the shoulder. Further, although the test setup was based on ASTM F2028, it is not a complete representation of the physiological loading processes in the glenohumeral joint.

## Conclusion

The medial irreversible displacement is the dominant motion component in bone-block reconstruction after critical bone loss of the anterior glenoid. The spina-scapula and coracoid bone blocks are comparable in terms of primary stability and extent of micromotion. Thus, spina-scapula bone blocks may be alternatives in bony glenoid reconstruction from a biomechanical point of view. However, further biomechanical studies with larger sample sizes are necessary to verify the presented results.

## Data Availability

The datasets used and/or analyzed during the current study are available from the corresponding author on reasonable request.
